# Real Life Cancer Comorbidity in Greek Patients with Diabetes Mellitus Followed Up at a Single Diabetes Center: An Unappreciated New Diabetes Complication

**DOI:** 10.1155/2014/231425

**Published:** 2014-07-22

**Authors:** Anastasia Thanopoulou, Demetrios Pectasides

**Affiliations:** Diabetes Centre, 2nd Department of Internal Medicine, National University of Athens, Hippokration General Hospital, 114 Vassilissis Sofias Avenue, 115 27 Athens, Greece

## Abstract

We determined cancer comorbidity in patients with diabetes followed up at a single Greek academic clinic and investigated the potential related factors. Cancer comorbidity was prospectively recorded for all patients with type 2 (T2DM, *n* = 759) or type 1 (T1DM, *n* = 134) diabetes of at least 10-year duration examined during one year. Patient characteristics, diabetes age of onset, duration, treatment, control, and complication rates were compared between subjects with and without cancer. Moreover, a retrospective collection of data from similar patients examined for the first time during the last 25 years, but lost to follow-up, after at least one-year's regular visits, was performed. In regularly followed-up T2DM patients cancer comorbidity was 12.6%. Patients with cancer were older and more frequently smokers. Prostate cancer was the most frequent (24.0%) type. In T1DM cancer comorbidity was 3.0%. Similar rates of comorbidity and types of cancer were observed in lost to follow-up patients. In conclusion, our patients with T2DM of at least 10-year' duration show high cancer comorbidity. No specific characteristics discriminate patients with cancer. Therefore presymptomatic cancer detection and prevention strategies may have to be incorporated into the annual systematic evaluation of our patients.

## 1. Introduction

Type 2 diabetes (T2DM) mellitus is highly prevalent and tends to become a “global epidemic.” The increase in T2DM incidence is in parallel to the “twin” “global pandemic” of obesity and is mainly attributed to it [1]. Both obesity and T2DM are independently associated with an increased risk of developing cancer and an increased mortality [2, 3]. In the interplay between obesity, T2DM, and cancer, insulin resistance and hyperinsulinemia are important determinants [4]. Insulin and insulin-like growth factors are well known as key regulators of metabolism and growth, affecting cell proliferation, differentiation, and apoptosis, mechanisms via which they are thought to be involved in the oncogenesis process [5]. Both T2DM and cancer have been associated with exposure to common environmental factors, such as high consumption of energy and animal fat and low consumption of fiber as well as low physical activity. These environmental factors are involved in the development of insulin resistance and compensatory hyperinsulinemia, and thus they influence the risk for both diabetes and cancer evolution [6].

The relation between T2DM and malignancies has been mostly studied in the form of the comparison of cancer development risk between subjects with diabetes and without diabetes. Subjects with type 2 diabetes mellitus (T2DM) have been found at an increased risk of cancer development compared to subjects without diabetes, even after adjustment for age, BMI, smoking, nutritional habits, and physical activity [2, 7].

Unlike T2DM, studies reporting on the association between type 1 diabetes (T1DM) and cancer risk are scanty. T1DM has been associated with a modest excess cancer risk overall and risks of specific cancers that differ from those associated with T2DM. In a Swedish study, patients with T1DM had elevated risks of cancers of the stomach, cervix, and endometrium [8]. In another study, ovarian cancer risk was found to be highly significantly raised in patients with T1DM diagnosed at ages 10–19 years [9].

Unfortunately, in Greece no reliable data on cancer incidence exist and, therefore, cancer morbidity cannot be easily compared between Greek subjects with or without diabetes.

During regular follow-up in the outpatient diabetes clinic, the authors had the impression that the frequency of malignancies appeared very high. Therefore, the present study was conducted to ascertain cancer comorbidity in our patients and to investigate potential related factors.

## 2. Patients and Methods

Data of all patients older than 20 years of age, having T2DM or T1DM of at least 10-year duration, was prospectively collected during their regular follow-up visits during one-year period. This relatively long diabetes duration was selected in order to ensure adequate exposure of the subjects to the diabetic milieu. The study was approved by the institutional ethics committee (number of approval ES 10/29) and had no funding.

Overall, 759 subjects with T2DM and 134 subjects with T1DM were included.Cancer comorbidity was recorded, as well as the type of cancer and the year of its diagnosis. Patients with diabetes onset after cancer diagnosis, as well as patients with drug-induced diabetes, were excluded. Patients with T2DM and T1DM were analyzed separately. Sex, age, age of diabetes onset, diabetes duration, anthropometrical data (body weight, height, BMI, and waist circumference), blood pressure levels, smoking status, antidiabetic medication, glycemic control, and frequency of diabetes complications were studied.

After analysis of data from regularly followed-up subjects, we also performed a retrospective collection of data from patients with the same characteristics, examined for the first time in our outpatient clinic during the last 25 years, but lost to follow-up after at least one-year regular visits. Cancer comorbidity, type of cancer, and year of cancer diagnosis were recorded wherever information was available. This was done in order to assess the already known cancer comorbidity. Naturally, no comparisons between subjects with or without cancer could be conducted in this cohort, since the real cancer comorbidity in subjects lost to follow-up might have been higher, due to cancer development after the patients' last visit to the clinic.

Statistical analysis was carried out by the statistical package for social sciences (SPSS, Chicago, IL) version 15. Differences between subjects with and without cancer were evaluated with Student's* t*-test for continuous variables with normal distribution and with* x* ^2^ test for categorical variables. A* p* correction for multiple comparisons was performed. Nonparametric tests were used where appropriate.

## 3. Results

Mean age of T2DM patients was 68.7 years, mean diabetes duration 19.6 years, mean BMI 28.5 kg/m^2^, and mean HbA_1c_ 7.0%. The corresponding values for T1DM patients were age of 46.5 years, mean diabetes duration of 23.1 years, mean BMI of 25.6 kg/m^2^, and mean HbA_1c_ of 7.5%. Most of the T2DM patients were on oral antidiabetic drugs (OADs, 59.2%), while 9.3% were treated with OADs plus basal insulin and 31.5% were exclusively treated with insulin. Current smokers or exsmokers were 52.7% of T2DM and 47.9% of T1DM patients. Cancer comorbidity was observed in 12.6% of T2DM patients. Mean age of cancer diagnosis was 65.2 ± 1.1 and cancer was diagnosed after 14.0 ± 1.0 years of diabetes duration. On the other hand, cancer comorbidity was observed in 3.0% of T1DM patients. The mean age of cancer development in T1DM patients was 46.5 ± 9.5 years.

The characteristics of patients with cancer in comparison to those without cancer are shown in Table 1. In T2DM subjects, patients with cancer were older, were diagnosed with diabetes at an older age, and were more frequently present or past smokers compared to patients without cancer. They did not differ in sex, anthropometric measurements, blood pressure levels, glycemic control, antidiabetic regime, duration of insulin therapy, or coexistence of diabetes complications. No difference in cancer comorbidity was observed between patients who developed T2DM before or after their 50th year of age or between patients who had T2DM duration lower or greater than 20 years. Similarly, no difference in cancer comorbidity was observed between normal weight, overweight, or obese T2DM patients (data not shown). Finally, no difference in cancer comorbidity was observed between patients taking or not taking metformin and patients on insulin therapy (including insulin glargine) compared to patients not on insulin therapy.

Cancer types in subjects with T2DM regularly followed up are shown in Table 2. Prostate cancer was the most frequently observed type of cancer in our patients (24.0%), followed by breast cancer, and so forth. Surprisingly, colon cancer was not common in our patients. The same was true for lung cancer, despite the high number of present smokers and exsmokers. In T1DM patients there were two breast cancers, one astrocytoma, and one case of Hodgkin's disease. All subjects developed cancer before the 14th year of diabetes duration.

After finding this considerably high comorbidity of cancer in our systematically followed-up patients, we retrospectively examined which was the rate of known cancer, in other patients followed up in our center in the past, but lost to follow up at present. These patients were examined for the first time in our outpatient clinic during the last 25 years and in order to be selected they had to have at least 10 years diabetes dutration. There were 950 T2DM patients with a cancer comorbidity of 10.6% and a mean age of cancer development of 65.7 ± 0.1 years and 102 T1DM patients with a cancer comorbidity of 2.0% (referring only to breast cancer) and a mean age of cancer development of 56.5 ± 10.5 years. The lost to follow-up patients' characteristics are shown in Table 3. The types of cancer in T2DM patients lost to follow-up are shown in Table 4.

They had mean diabetes duration (18.0 ± 0.2 years), BMI (27.9 ± 0.1 kg/m^2^), and HbA_1c_  7.5 ± 0.0%, very similar to regularly followed-up subjects. Moreover, most of them were also on OADs (68.6%), while 2.3% were on OADs plus basal insulin and 29.1% were exclusively insulin treated. Prostate cancer was the most frequently observed type (14.9%), as it was for the regularly followed-up patients, followed by breast, urinary tract, and colon cancer (12.9% for each), hematological malignancies, and so forth.

## 4. Discussion

The present study is the first one, to the best of our knowledge, to outline the “real life” problem of cancer comorbidity among systematically followed-up patients in a single outpatient diabetes clinic in Greece. Patients with at least 10 years of diabetes duration were selected, in order to have adequate exposure to the diabetic state and thus exhibit possible effect of diabetes on oncogenicity, which is well known to be a long-lasting procedure. Some studies have shown an association between newly diagnosed diabetes and especially pancreatic cancer [10], but, in that case, diabetes should be considered a result, that is, a clinical manifestation of cancer, than a causal factor.

Cancer comorbidity of 12.6% in regularly followed-up and of 10.6% in lost to follow-up T2DM patients was observed in our study. Moreover much lower cancer comorbidity (3.0% in regularly followed-up and 2.0% in lost to follow-up patients) was observed in T1DM patients, who were of course much younger. Unfortunately data on cancer prevalence are lacking in Greece, so the cancer comorbidity of our study population cannot be compared with that of the general population. By any means, this was not the aim of our study, since the knowledge that cancer is more prevalent in subjects with diabetes seems to be supported by several lines of evidence.

Patients with diabetes are an extremely heterogeneous group in terms of glycemic control, coexistence of diabetes complications, medication used, diet, degree of obesity, and endogenous insulin levels.Systemically followed-up patients in the same center are of course individually treated but, more or less, treated according to the same therapeutic perception. This diminishes discrepancies in the clinical course of the patients caused by exogenous factors and whatever happens to them can only be attributed to each individual patient's particularities (biological, etc.). In the present study no difference in metformin or insulin use was observed between patients with or without cancer.

In the present study, no difference in BMI was observed between subjects with and without cancer, both for T1DM and T2DM. Moreover, no difference in cancer comorbidity was observed between normal weight, overweight, and obese T2DM subjects. These findings, merely referred to in the context of the results of the present study, do not seem to have a special interpretation and should not take anyone's thought off the fact that being overweight is second only to smoking as a clear and avoidable cause of cancer [11].

The association between smoking and the development of cancer in general and especially of some types of cancer is well documented [12]. The same is true for the association between smoking and cardiovascular disease [13]. This is the reason why smoking cessation is promoted and instructed with emphasis in our center in all smoking patients, at every visit. Nevertheless, many of our patients are current smokers or exsmokers. As it can be presumed, the percentage of smokers was significantly higher in T2DM patients with cancer in comparison to those without cancer. These two facts testify the urgent need to persuade and help patients quit smoking.

HbA_1c_ levels and the presence of the classical diabetes complications did not differ between subjects with or without cancer, at least in our population, showing possibly that cancer development in patients with diabetes is not associated with the severity of diabetes, but with something else, such as the already known common for the two conditions risk factors or something unknown till present. Of course one must not forget that every unexplained worsening of diabetes control might be an alert signal for cancer development.

The most common type of malignancy in our patients was prostate cancer, although in the literature diabetes is associated with reduced risk of prostate cancer [14], mainly due to lower serum testosterone concentration found in diabetic men compared to nondiabetic [15]. The findings of our study are in line with those of Veterans Integrated Services Network study, where, in 87,678 male patients with diabetes, cancer comorbidity was 11.3% in total, almost as it was in our study, and prostate cancer was the most frequently observed type [16].

Breast cancer, which was the second cancer in frequency in T2DM patients and the most frequent cancer in T1DM patients in our study, is referred in most but not all studies to be associated with diabetes, especially in postmenopausal women, with a varying relative risk in different studies and a 1.20 relative risk found in a meta-analysis [17].

Colon cancer was not common in our patients (8.3%). Colorectal carcinogenesis is a model of diabetes related oncogenesis and is associated with hyperinsulinemia. Both diabetes and colon cancer development have been attributed to exposure to common risk factors such as energy dense, high fat, low fibre diets, and physical inactivity [18].

Lung cancer, the most common cancer in Southern Europe, was not frequent in our patients (5.2%), despite the great number of present smokers and exsmokers. A study based on Great Britain's General Practice Research Database, with more than 66,000 participants with diabetes, does not also confirm a greater risk for lung cancer in patients with diabetes than in the general population [19].

Our lost to follow-up patients, till the time they were last examined, had very similar characteristics to our regularly followed-up patients. Their cancer comorbidity was also very similar, strengthening the value of our observation, since it refers to 1,709 patients with T2DM and 236 patients with T1DM of at least 10-year duration, followed up at a single center.

Whereas cardiovascular complications are well known for diabetes and all our efforts focus on their prevention, the frequent coexistence of diabetes and cancer does not seem to have been consolidatedin the thought of doctors treating subjects with diabetes. On the other hand, subjects with diabetes undergo yearly full clinical and laboratory examination for the detection of the possible emergence of “classical” complications. Because of the amplitude of this control, many patients derive, unfortunately, the wrong impression that they have undergone all presymptomatic control necessary for their health [20]. The question is whether frequent coexistence of diabetes with malignancies should be considered as a “new,” unappreciated till now, diabetes complication. In that case, doctors dealing with diabetes will strengthen their efforts to educate their patients in the strategies of cancer prevention, the way they do for cardiovascular diseases' prevention strategies. It is noteworthy that these strategies have a lot in common (prevention of obesity, increase of physical activity, choice or rejection of certain foodstuff, etc.). Also they will probably include in the annual patients' evaluation the acceptable examinations for presymptomatic cancer detection, depending on each patient's sex, age, and familial anamnesis.

In conclusion, patients with T2DM of at least 10-year duration regularly followed up at our clinic exhibit highcancer comorbidity, while patients with T1DM do not. Importantly, no specific characteristics can discriminate patients with cancer; therefore, presymptomatic cancer detection and prevention strategies may have to be reinforced and incorporated into the annual systematic evaluation of subjects with diabetes.

## Figures and Tables

**Table 1:** Characteristics of regularly followed-up subjects with cancer in comparison to subjects without cancer.

	T2DM			T1DM		
	CA + (*n* = 96) 12.6%	CA − (*n* = 663)	*P*	CA + (*n* = 4) 3.0%	CA − (*n* = 127)	*P*
Sex (males)	63.5%	57.9%	NS	50.0%	50.0%	NS
Age	70.9 ± 1.0	68.3 ± 0.4	<0.05	49.0 ± 9.0	46.4 ± 1.3	NS
Age of diabetes onset	51.2 ± 1.1	48.9 ± 0.4	<0.05	37.7 ± 8.7	23.4 ± 1.2	<0.05
Diabetes duration (years)	20.8 ± 0.9	19.5 ± 0.3	NS	11.5 ± 0.6	23.5 ± 0.9	<0.05
Smoking	66.7%	50.8%	<0.01	50.0%	47.9%	NS
BMI	28.5 ± 0.4	28.5 ± 0.2	NS	30.2 ± 2.2	25.5 ± 0.3	NS
Weight (kg) (males)	79.9 ± 1.5	80.1 ± 0.6	NS	95.5 ± 7.5	80.0 ± 1.3	NS
Weight (kg) (females)	75.8 ± 1.7	73.7 ± 0.9	NS	75.0 ± 18.0	65.3 ± 1.4	NS
Height (m) (males)	1.70 ± 0.0	1.70 ± 0.0	NS	1.83 ± 0.1	1.80 ± 0.0	NS
Height (m) (females)	1.58 ± 0.0	1.58 ± 0.0	NS	1.52 ± 0.0	1.60 ± 0.0	NS
Waist (cm) (males)	99.8 ± 1.2	99.1 ± 0.9	NS	101.2 ± 5.1	98.9 ± 1.3	NS
Waist (cm) (females)	95.8 ± 1.6	96.5 ± 0.3	NS	97.9 ± 1.1	87.6 ± 1.1	NS
SAP (mmHg)	138.2 ± 1.4	133.6 ± 1.5	NS	130.3 ± 1.0	129.6 ± 1.2	NS
DAP (mmHg)	79.6 ± 1.2	74.8 ± 0.8	NS	73.2 ± 0.2	75.8 ± 0.6	NS
A1c%	7.0 ± 0.1	7.0 ± 0.0	NS	8.1 ± 0.4	7.5 ± 0.4	NS
OADs (%)	53.1%	60.0%	NS			
OADs + basal insulin (%)	13.5%	8.7%	NS			
Insulin treated (%)	33.3%	31.2%	NS			
Years of diabetes before insulin initiation	16.3 ± 1.2	14.9 ± 0.5	NS			
Duration of insulin therapy (years)	7.2 ± 1.0	7.2 ± 0.4	NS			
“Classical” diabetes complications coexistence (%)	57.1%	68.8%	NS	66.7%	47.4%	NS

OADs: oral antidiabetic drugs.

**Table 2:** Types of cancer in subjects with T2DM regularly followed up.

	*n*	%
Urinary tract		
Prostate	23	24.0
Bladder	7	7.3
Kidney	2	2.1
Breast		
Breast	14	14.6
Hematological malignancies		
Lymphomas	6	6.3
Leukemias	3	3.0
Gastrointestinal		
Colon	8	8.3
Pancreas	2	2.1
Stomach	1	1.0
HCC	1	1.0
Cholangiocarcinoma	1	1.0
GIST	1	1.0
Lung		
Lung	5	5.2
Female genitals		
Endometrial	3	3.1
Ovarian	3	3.1
Thyroid		
Thyroid	6	6.3
Skin		
Nonmelanoma	3	3.1
Melanoma	2	2.1
Rest		
Larynx	2	2.1
Pharynx	1	1.0
Buccal	1	1.0
Chondrosarcoma	1	1.0
Total	**96**	**100.0**

**Table 3:** Data of all patients with at least 10-year diabetes duration examined for the first time during the last 25 years but lost to follow-up after at least one-year regular visits.

*N*	T2DM		T1DM	
	950 (All)	CA + (n = 101)	102 (all)	CA + (n = 2)
		10.6%		2.0%
Sex (male)	545 (57.4%)	54 (54.5%)	61 (59.8%)	
Age	67.9 ± 0.3	69.7 ± 0.9	41.6 ± 1.4	57.5 ± 9.5
Age of diabetes onset	50.2 ± 0.3	53.0 ± 1.1	21.7 ± 1.4	25.5 ± 7.5
Diabetes duration	18.0 ± 0.2	19.3 ± 0.9	19.9 ± 0.9	32.0 ± 2.0
OADs (%)	652 (68.6%)	63.4%		
OADs + basal insulin	22 (2.3%)	1.0%		
Insulin treated	276 (29.1%)	35.6%		
Years of DM before insulin initiation	15.1 ± 0.4	13.9 ± 1.2		
A1c	7.5 ± 0.0	7.2 ± 0.1	8.4 ± 0.2	6.5 ± 1.2
BMI	27.9 ± 0.1	28.0 ± 0.5	25.5 ± 0.4	22.6 ± 0.0

OADs: oral antidiabetic drugs.

**Table 4:** Types of cancer in T2DM patients lost to follow-up.

Types of cancer (*n* = 101)	Frequency order of the cancer	%
	type in the regularly	
	followed-up T2DM population	
Prostate cancer	1	14.9%
Breast cancer	2	12.9%
Urinary tract	3	12.9%
Colon cancer	5	11.9%
Hematological malignancies	4	7.0%
Pancreatic cancer	10	6.9%
Female genital cancer	7	6.0%
Skin cancer	9	5.0%
Lung cancer	6	3.0%
Thyroid cancer	8	3.0%
Rest of GI; rest of URT; unknown		16.5%
